# A Method of Rendering Iodoform Inodorous

**Published:** 1886-09

**Authors:** C. K. Gregg

**Affiliations:** Ramos Arispe, Mexico


### A METHOD OF RENDERING IODOFORM INODOROUS.

*By C. K. Gregg, M. D. Ramos Arispe, Mexico.*

[For Daniel's Texas Medical Journal.]

I WOULD like to call the attention of the profession to a mode of disguising completely, the disagreeable odor of a drug which we are called upon so frequently to use, and whose good effects are so marked. Having had quite a large amount of venereal practice among the natives and employees of the Mexican National R. R., I have been endeavoring for some time to discover some means of improving the odor of our "sheet anchor" remedy in syphilis and chancroids with their complications and sequelæ; also acute and chronic ulcers of a non-specific type. The odor of the drug in some instances, especially to the Mexicans, who have very delicate stomachs, being so powerful as to cause nausea and even vomiting—and also making them ashamed to mix socially with their friends. Hence it seems absolutely necessary that we should devise some means of evading such baneful effects of a local application so efficient. In the May number of the *Journal of Cutaneous*

*and Venereal Diseases* a long list of drugs for rendering Iodoform-inodorous is mentioned, and none of which it is stated have proved efficient. However I commenced some time ago to use oil of peppermint, and with the happiest results. By means of 5 drops of the oil to one ounce of the dry powder or ointment, I have found that the odor is rendered entirely inocuous. Patients who are not always careful and keep the air excluded, do not complain of the odor returning by such exposure. I have tried this procedure in a great many cases and have never had any complaints offered. I trust a worthy trial will be given to this simple method, for if it proves a success in the hands of others, we have accomplished much in the mode of treating those loathsome local lesions with our most potent remedy.

